# A Novel *In Vivo* Infection Model To Study Papillomavirus-Mediated Disease of the Female Reproductive Tract

**DOI:** 10.1128/mBio.00180-19

**Published:** 2019-03-05

**Authors:** Megan E. Spurgeon, Aayushi Uberoi, Stephanie M. McGregor, Tao Wei, Ella Ward-Shaw, Paul F. Lambert

**Affiliations:** a McArdle Laboratory for Cancer Research, University of Wisconsin School of Medicine and Public Health, Madison, Wisconsin, USA; b Department of Pathology and Laboratory Medicine, University of Wisconsin School of Medicine and Public Health, Madison, Wisconsin, USA; University of Michigan-Ann Arbor; University of South Carolina School of Medicine; Kansas State University

**Keywords:** cancer, cervix, infectious disease, mouse, papillomavirus

## Abstract

Papillomaviruses exhibit species-specific tropism, thereby limiting understanding and research of several aspects of HPV infection and carcinogenesis. The discovery of a murine papillomavirus (MmuPV1) provides the opportunity to study papillomavirus infections in a tractable, *in vivo* laboratory model. MmuPV1 infects and causes disease in the cutaneous epithelium, as well as the mucosal epithelia of the oral cavity and anogenital tract. In this report, we describe a murine model of MmuPV1 infection and neoplastic disease in the female reproductive tracts of wild-type immunocompetent FVB mice. Low-grade dysplastic lesions developed in reproductive tracts of FVB mice infected with MmuPV1 for 4 months, and mice infected for 6 months developed significantly worse disease, including squamous cell carcinoma (SCC). We also tested the contribution of estrogen and/or UV radiation (UVR), two cofactors we previously identified as being involved in papillomavirus-mediated disease, to cervicovaginal disease. Similar to HPV16 transgenic mice, exogenous estrogen treatment induced high-grade precancerous lesions in the reproductive tracts of MmuPV1-infected mice by 4 months and together with MmuPV1 efficiently induced SCC by 6 months. UV radiation and exogenous estrogen cooperated to promote carcinogenesis in MmuPV1-infected mice. This murine infection model represents the first instance of *de novo* papillomavirus-mediated carcinogenesis in the female reproductive tract of wild-type mice resulting from active virus infection and is also the first report of the female hormone estrogen contributing to this process. This model will provide an additional platform for fundamental studies on papillomavirus infection, cervicovaginal disease, and the role of cellular cofactors during papillomavirus-induced carcinogenesis.

## Abstract (precis)

Tractable and efficient models of papillomavirus-induced pathogenesis are limited due to the strict species-specific and tissue-specific tropism of these viruses. Here, we report a novel preclinical murine model of papillomavirus-induced cervicovaginal disease in wild-type, immunocompetent mice using the recently discovered murine papillomavirus, MmuPV1. In this model, MmuPV1 establishes persistent viral infections in the mucosal epithelia of the female reproductive tract, a necessary component needed to accurately mimic HPV-mediated neoplastic disease in humans. Persistent MmuPV1 infections were able to induce progressive neoplastic disease and carcinogenesis, either alone or in combination with previously identified cofactors of papillomavirus-induced disease. This new model will provide a much-needed platform for basic and translational studies on both papillomavirus infection and associated disease in immunocompetent mice.

## INTRODUCTION

Viruses cause approximately 15% of all human cancers globally (1), and high-risk human papillomaviruses (HPVs), a group of small DNA tumor viruses that infect the stratified squamous epithelia, are alone responsible for nearly 5% of human cancers (2). HPVs are the most common sexually transmitted infection in the United States (3). There are ∼40 mucosotropic HPV genotypes that preferentially infect the mucosal, stratified epithelia of the anogenital tract (cervix, vagina, and anus) and the oral cavity (4, 5). Nearly all cervical cancers are caused by high-risk mucosotropic HPVs (e.g., HPV16, -18, and -31) (6, 7). Most HPV infections are transient, but persistent infection with high-risk genotypes can lead to the progression of precancerous lesions to invasive cancer (8, 9). Although HPV vaccines protect against new high-risk HPV infections, they have no effect on preexisting infections and are largely unavailable in developing nations where HPV-associated cervical cancer is often the leading cause of death by cancer in women (10, 11). Consequently, there remains an urgent need to explore more effective and accessible therapeutic interventions. Meeting this need would be greatly facilitated by the availability of a preclinical model in which papillomavirus infection results in cervical cancer.

Papillomaviruses exhibit strict species- and tissue-specific tropism, characteristics that have long hindered the ability to study aspects of papillomavirus infection and disease in an efficient laboratory model. While several models have been developed in rodents and primates (12–15), these models are often costly and onerous due to the scarcity of available genetically modified hosts and reagents (15, 16). Recently, a murine papillomavirus (MmuPV1 or MusPV1) was isolated from skin papillomas arising in T-cell-deficient, hairless *FoxN1^nu/nu^* (nude) mice (17). MmuPV1 presents a valuable animal virus to study papillomavirus infection and disease in laboratory mice, which are tractable, genetically manipulable, and relatively affordable (17). MmuPV1 infects both cutaneous and mucosal epithelia and subsequently promotes disease and neoplastic progression (18–24). While MmuPV1 has been largely studied thus far in the context of cutaneous disease (17, 22, 24–27), MmuPV1 exhibits expanded tissue tropism and causes disease in the female reproductive tracts of *FoxN1^nu/nu^* mice (18) and carcinoma *in situ* in *FoxN1^nu/+^* mice (28). We recently reported that UV radiation (UVR) makes wild-type, immunocompetent mice highly susceptible to MmuPV1-induced cutaneous disease (24). Prior studies using HPV transgenic mouse models established a role for the female hormone estrogen in papillomavirus-mediated carcinogenesis in mucosal epithelia of the reproductive tract (29–31) and demonstrated that estrogen and its nuclear receptor, estrogen receptor alpha (ERα), are necessary cofactors required for the genesis and maintenance of cervical carcinogenesis (31–33). We therefore sought to determine if MmuPV1 causes neoplastic disease in the female reproductive tract of wild-type, immunocompetent mice and whether exogenous estrogen and UVR, alone or together, contribute to neoplastic progression.

While MmuPV1 has been reported not to cause cutaneous disease efficiently in mice with intact immune systems (17, 24, 26, 27), we found that infection with MmuPV1 induced formation of low-grade precancerous lesions in the female lower reproductive tracts of wild-type immunocompetent animals after 4 months. Treatment of MmuPV1-infected mice with either UVR or estrogen led to high-grade precancerous lesions, and treatment with both UVR and estrogen led to an increased burden of precancerous lesions as well as cervicovaginal cancers. Allowing MmuPV1 infection to proceed for 6 months, either alone or in combination with exogenous estrogen treatment, was sufficient to promote the development of high-grade lesions and cancers. This is the first report of *in vivo* cervical carcinogenesis induced by a natural papillomavirus infection in immunocompetent mice, and these results provide a novel preclinical model system for basic and translational studies on papillomavirus-induced cervicovaginal disease.

## RESULTS

### Development and histopathological scoring of an MmuPV1 infection model in the murine female reproductive tract.

**FIG 1:**
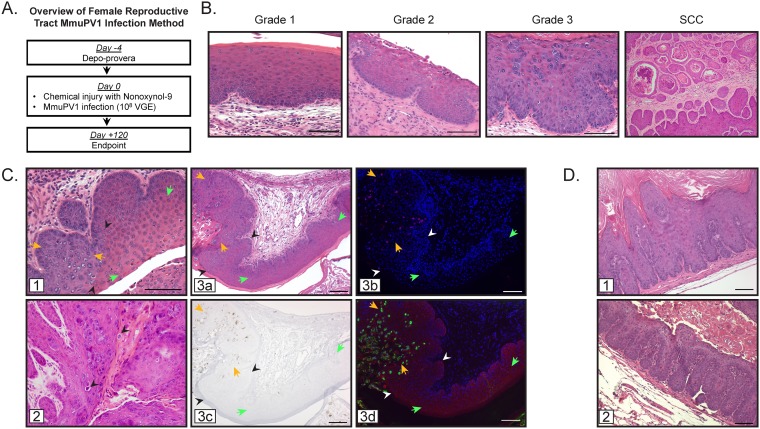
Development and histopathological scoring of an MmuPV1 infection model in the murine female reproductive tract. (A) Method for MmuPV1 infection of the murine female reproductive tract in FVB mice. Four days prior to infection (day −4), mice were treated with medroxyprogesterone acetate (Depo-Provera). On day 0, mice were treated with nonoxynol-9 4 h prior to infection with approximately 10^8^ VGE of MmuPV1. Infection was allowed to proceed for 4 months (day 120). (B) Representative H&E-stained tissue sections from FVB mice showing histopathology of progressive neoplastic disease grades 1 to 3 and SCC in MmuPV1-infected female reproductive tracts. (C) Notable histological features in the MmuPV1-infected reproductive tract. Junctions between uninfected and MmuPV1-infected cervicovaginal epithelia (panel 1; black arrowheads indicate junction between uninfected [green arrows] and infected [orange arrows] epithelia) and the presence of koilocytes in infected regions (panel 2; black arrowheads). Productively infected regions (panel 3a; junction between uninfected [green arrows] and infected [orange arrows] epithelia indicated with black/white arrowheads) were verified as being MmuPV1 positive by staining for MmuPV1 DNA by FISH (panel 3b; pink, MmuPV1 DNA; blue, DAPI nuclear stain), MmuPV1 transcript E1^E4 by RNAscope (panel 3c; brown, E1^E4 transcript; blue, hematoxylin counterstain), and MmuPV1 capsid protein L1 by indirect immunofluorescence (panel 3d; red, MmuPV1 L1; green, keratin; blue, DAPI nuclear stain). (D) Additional histological features observed during MmuPV1-associated neoplastic progression include exophytic morphology (panel 1) and extensive papillation into the underlying stroma (panel 2). All scale bars = 100 μm.

To infect the lower female murine reproductive tract with MmuPV1 (Fig. 1A), we adapted a cervicovaginal infection method originally developed by Roberts and colleagues (34). We have previously used this same method to infect mice with HPV pseudovirus (35). Female mice were first treated with medroxyprogesterone acetate (Depo-Provera) to synchronize mice in diestrus and subsequently with nonoxynol-9 (N-9), causing chemical injury to the cervicovaginal epithelium to potentiate papillomavirus entry (34). Mice were then infected in the cervicovaginal canal with 10^8^ VGE (viral genome equivalents) MmuPV1. Four to six months postinfection, reproductive tracts were harvested and histopathologically evaluated.

Our well-established scoring system, described in detail by Riley et al. (31), formed the framework that guided the overall descriptions of neoplastic disease in the novel MmuPV1 infection model (Fig. 1B). We have traditionally used these histopathological scoring criteria to grade progressive neoplastic disease in estrogen-treated HPV transgenic mice (29–32), in which expression of the HPV16 oncogenes E6 and/or E7 is directed to basal cells of stratified epithelia using the keratin 14 (K14) promoter (36, 37). The MmuPV1 infection model involves disease caused by an active, replicating virus, thus introducing additional pathological considerations. Criteria used to score neoplastic disease include tissue organization/architecture, degree of nuclear atypia, and presence or absence of the basement membrane. During the course of neoplastic progression through successive grades of intraepithelial neoplasia, there are corresponding increases in tissue disorganization and architectural complexity, involving expansion of the basal cell layer composed of epithelial cells with higher nuclear/cytoplasmic ratio, and increased invaginations of the basement membrane into the underlying stroma. Squamous cell carcinomas (SCC) achieve all of these criteria and can also include invasive cancer cell clusters, some of which are associated with keratin pearls.

In scoring MmuPV1-infected reproductive tracts, the separate cervical and vaginal scores were combined into one cervicovaginal score, as most dysplastic lesions arose on a continuous epithelial surface correlating with exposure to the virus inoculum. Cytological effects of the virus are evident in the reproductive tract, which is a notable difference from histopathological features of HPV16 transgenic models that do not involve natural infection. Focal areas of MmuPV1 infection resulted in clear boundaries between epithelium with normal histopathology and regions of epithelia with histological hallmarks of virus infection (Fig. 1C, panels 1 and 3a), including karyomegaly, accumulation of amphophilic to basophilic cytoplasm, occasional perinuclear “halos” akin to koilocytes characteristic of human papillomavirus infection, and dense homogeneous chromatin similar to that seen in inclusions of other viruses (Fig. 1C, panel 2). The regions displaying histological signs of infection were verified as being associated with MmuPV1 infection, as shown by positive staining for amplified viral DNA (Fig. 1C, panel 3b), viral E1^E4 mRNA transcripts (Fig. 1C, panel 3c), and expression of the viral major capsid protein L1 (Fig. 1C, panel 3d). Similarly to SCC observed in HPV16 transgenic mice, SCC in the infected mice demonstrated keratinization in the form of extensive keratin pearls and accumulation of keratinaceous debris in microcysts (Fig. 1B, far right panel). SCC in the infected mice predominantly demonstrated a degree of architectural complexity exceeding characteristics of a dysplastic process and that are better regarded as carcinomas with a pushing border, though occasional foci of frank invasion in the form of small invasive clusters were also identified. A spectrum of histological changes was evident in MmuPV1-infected mice, and keratin pearls were also present in cases lacking sufficient complexity to be regarded as carcinoma. There were also areas exhibiting exophytic morphology (Fig. 1D, panel 1) and intense papillation into the underlying stroma (Fig. 1D, panel 2). These additional characteristics were incorporated into our previously established scoring guide in order to analyze MmuPV1-induced disease and neoplastic progression.

### MmuPV1 infects and causes neoplastic disease in the murine female reproductive tract of immunocompetent mice alone and in combination with UVB and estrogen treatment.

We sought to verify if our adapted infection protocol results in infection of the cervicovaginal tract in 6-week-old immunodeficient *FoxN1^nu/nu^* mice infected with 10^8^ VGE MmuPV1 for 4 months. Consistent with previous reports (18, 28), we found that MmuPV1 establishes a productive virus infection in the mucosal epithelia of the lower reproductive tract of immunodeficient mice and results in neoplastic disease (see Fig. S1 in the supplemental material). All MmuPV1-infected *FoxN1^nu/nu^* mice (*n* = 9) developed neoplastic disease in the cervicovaginal epithelia, and 44% (*n* = 4/9) developed SCC. These results demonstrated that our protocol for female reproductive tract infection results in productive MmuPV1 infection, thus establishing a platform for additional studies in wild-type, immunocompetent FVB mice.

10.1128/mBio.00180-19.1 FIG S1 MmuPV1 infection causes neoplastic disease in the murine female reproductive tract of immunodeficient mice. (A) Representative H&E-stained tissue sections from MmuPV1-infected immunodeficient *FoxN1^nu/nu^* female mice showing high-grade dysplastic disease present at 4 months postinfection. (B) H&E-stained image of cervicovaginal epithelia of *FoxN1^nu/nu^* female mice (left) and indirect immunofluorescence for MmuPV1 L1 protein (green, L1; red, keratin; blue, DAPI). The epithelial-stromal boundary is indicated with the white dashed line. (C) Cervicovaginal disease severity in the female reproductive tracts of MmuPV1-infected *FoxN1^nu/nu^* mice (*n* = 9) was determined using histopathological analysis. The percentage of mice with each disease state is indicated (top). Disease severity by anatomical location in MmuPV1-infected *FoxN1^nu/nu^* mice, with the number of mice with each indicated grade of disease in the cervix (black bars) and vagina (gray bars) (bottom) shown. Download FIG S1, PDF file, 8.9 MB. Copyright © 2019 Spurgeon et al. 2019 Spurgeon et al. This content is distributed under the terms of the Creative Commons Attribution 4.0 International license.

**FIG 2:**
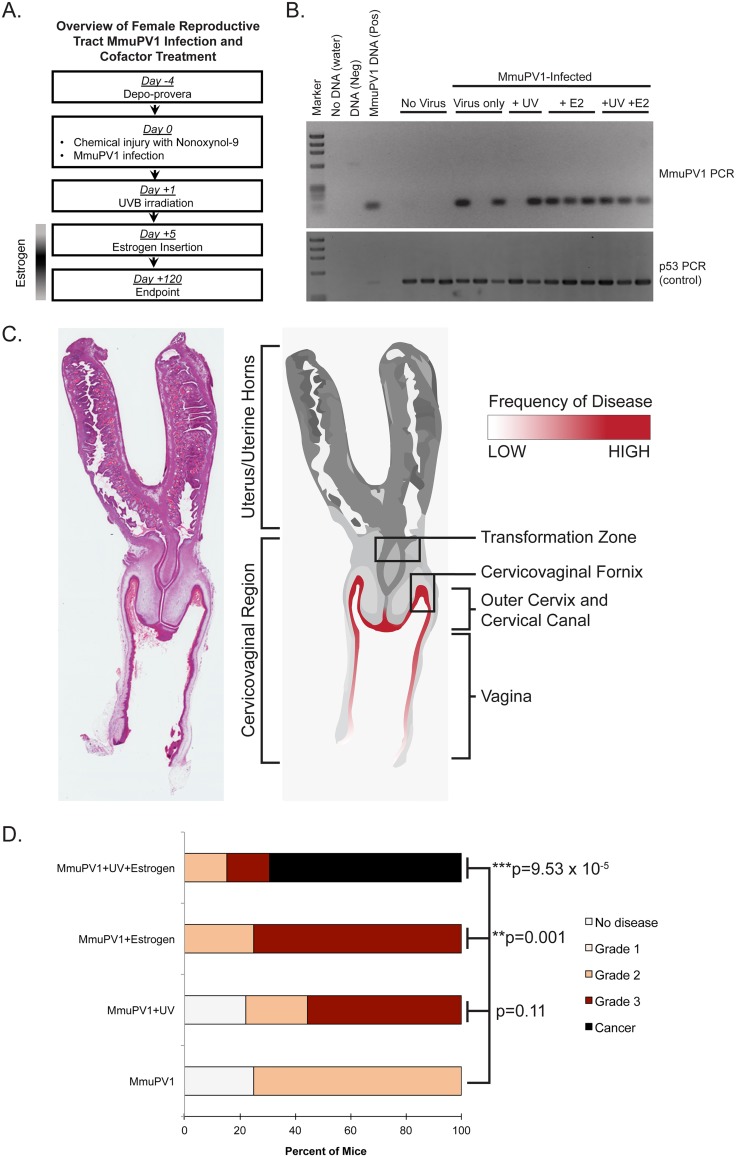
MmuPV1 infects and causes neoplastic disease in the murine female reproductive tract of immunocompetent mice alone and in combination with UVB and estrogen treatment. (A) Overview of MmuPV1 infection of female reproductive tract in FVB mice combined with cofactor treatment. When applicable, mice were irradiated with 1,000 mJ/cm^2^ UVB 1 day postinfection, and estrogen insertion was performed 5 days postinfection. (B) DNA was isolated from cervicovaginal lavage samples and analyzed by PCR for the MmuPV1 E2 gene (top) or for the p53 gene (bottom) to verify DNA presence/quality. (C) Anatomical location of MmuPV1-induced neoplastic disease development in the female reproductive tract of FVB mice. A full-slide scan of a representative H&E-stained murine female reproductive tract is shown on the left. A rendering of this reproductive tract is shown on the right. Regions of epithelia where disease developed in FVB mice are highlighted in red, where the intensity of red shading corresponds with the frequency of disease observed at each site. (D) Disease severity in cohorts of MmuPV1-infected mice as determined by histopathological analysis. Statistical analysis for overall disease severity was performed using a two-sided Wilcoxon rank sum test. For numbers of mice per group, please refer to Table 1.

We previously reported that UV B radiation (UVB) renders wild-type immunocompetent mice highly susceptible to MmuPV1-induced cutaneous disease (24). Estrogen has also been identified as a necessary cofactor for cervical carcinogenesis in HPV16 transgenic mouse models (29, 31–33). We therefore sought to determine whether MmuPV1 causes disease in the mucosal epithelia of the female reproductive tract in wild-type, immunocompetent FVB mice, either alone or in combination with UVB and/or exogenous estrogen. Combining our optimized infection methodology with additional cofactor treatments (Fig. 2A), we infected four different cohorts of immunocompetent female FVB mice with 10^8^ VGE of MmuPV1 virus for 4 months: (i) MmuPV1 only, (ii) MmuPV1-infected mice irradiated with 1000 mJ/cm^2^ UVB (MmuPV1+UV), (iii) MmuPV1-infected mice treated with exogenous estrogen (0.05 mg 17β-estradiol over 60 days) (MmuPV1+E2), and (iv) MmuPV1-infected mice that were both UVB irradiated and treated with estrogen (MmuPV1+UV+E2). Each cohort also included mock-infected controls.

To evaluate reproductive tracts for persistent MmuPV1 infection, cervicovaginal lavage samples were collected from infected mice at the study endpoint and tested for the presence of MmuPV1 DNA by adapting methods described previously (21, 28). In this study, a persistent MmuPV1 infection is defined as an infection that remains detectable, either by MmuPV1-specific PCR of cervicovaginal lavage samples or by directly staining positive for viral transcripts or capsid proteins in cervicovaginal tissues, several months after the mice are initially infected. Briefly, total DNA was isolated from lavage samples and amplified by PCR using primers specific to the MmuPV1 E2 gene (Fig. 2B). While we routinely observed variability in the presence of MmuPV1 DNA in mice infected with MmuPV1 only and MmuPV1+UV, we consistently detected viral DNA in the cervicovaginal lavage samples of mice in the MmuPV1+E2 and MmuPV1+UV+E2 cohorts. Pretreatment with medroxyprogesterone acetate was necessary to achieve persistent infections and subsequent disease in MmuPV1+UV+E2 mice (Fig. S2). These results indicate that MmuPV1 can establish persistent infections in the mucosal epithelia of wild-type, immunocompetent FVB mice.

10.1128/mBio.00180-19.2 FIG S2 Medroxyprogesterone acetate is required for optimal MmuPV1 infection and neoplastic progression in the female reproductive tract. (A) Disease severity comparison at 4 months postinfection in the female reproductive tract of FVB mice treated with (*n* = 13) or without (*n* = 7) medroxyprogesterone acetate prior to MmuPV1+UV+E2 infection. Overall disease severity was compared using a two-sided Wilcoxon rank sum test, and asterisks indicate *P* ≤ 0.001. (B) PCR analysis for the MmuPV1 E2 gene (top) on cervicovaginal lavage samples from MmuPV1+UV+E2 mice at 4 months postinfection that did or did not receive treatment with medroxyprogesterone acetate prior to MmuPV1 infection. PCR for the p53 gene (bottom) was performed to verify DNA presence/quality. (C) H&E and indirect immunofluorescence analysis for L1 (red), K14 (green), and DAPI (blue) on samples that were identified as negative or positive by cervicovaginal lavage (CVL) PCR analysis in panel B. The two samples identified as being positive (samples 5 and 6) in the no-medroxyprogesterone acetate group stained positive for MmuPV1 L1. All scale bars = 100 μm. Download FIG S2, PDF file, 6.2 MB. Copyright © 2019 Spurgeon et al. 2019 Spurgeon et al. This content is distributed under the terms of the Creative Commons Attribution 4.0 International license.

Reproductive tracts were initially harvested 4 months postinfection, and histopathological analysis was performed to score for neoplastic disease. To ascertain whether certain anatomical sites preferentially developed MmuPV1-induced disease, separate histopathological scores were first assigned separately for the vagina and cervix (Table 1), consistent with the scoring criteria for HPV16 transgenic mice (31). We found that most lesions developed in areas that were exposed to the virus inoculum, such as cervicovaginal fornices, outer cervix, outermost regions of the cervical canal, and the vaginal canal (Fig. 2C). The endocervix and transformation zone were rarely infected and rarely developed neoplastic lesions. The overall cervicovaginal disease severity was compared between each infected cohort and its mock-infected control cohort (Table 1). Disease severity in the MmuPV1-only and MmuPV1+UV groups was not significantly different from mock-infected counterparts (Mock versus MmuPV1 only, *P* = 0.219; Mock+UV versus MmuPV1+UV, *P* = 0.446). However, estrogen treatment significantly increased the severity of disease in MmuPV1+E2 mice (Mock+E2 versus MmuPV1+E2, *P* = 0.004). Likewise, overall disease severity was significantly higher in MmuPV1-infected mice treated with both UVB and exogenous estrogen (Mock+UV+E2 versus MmuPV1+UV+E2, *P* = 0.001). We observed SCC development only in MmuPV1+UV+E2-treated mice (*n* = 5/13 cervix, 38%; *n* = 7/13 vagina, 54%) at the 4-month endpoint. We found C567BL/6 mice infected with MmuPV1+UV+E2 to be resistant to MmuPV1-induced high-grade disease in the reproductive tract, and we were unable to detect any L1-positive cells 4 months postinfection in this strain of mice (Fig. S3).

**TABLE 1:** Summary of disease in FVB/N immunocompetent mice following MmuPV1 infection of the female reproductive tract^*a*^

Treatment group	Group size, *n*	No disease, cervix (vagina)	Dysplasia only			SCC cancer, cervix (vagina)
			CIN1 (VIN1)	CIN2 (VIN2)	CIN3 (VIN3)	
Mock	4	2 (2)	2 (1)	0 (1)	0 (0)	0 (0)
Mock+E2	3	0 (0)	3 (3)	0 (0)	0 (0)	0 (0)
Mock+UVB	3	1 (0)	2 (0)	0 (3)	0 (0)	0 (0)
Mock+UVB+E2	3	3 (3)	0 (0)	0 (0)	0 (0)	0 (0)
MmuPV1 Only	8	2 (2)	3 (0)	3 (6)	0 (0)	0 (0)
MmuPV1+E2	8	0 (0)	0 (0)	4 (2)	4 (6)	0 (0)*
MmuPV1+UVB	9	3 (2)	1 (0)	4 (3)	1 (4)	0 (0)
MmuPV1+UVB+E2	13	0 (0)	1 (1)	5 (1)	2 (4)	5 (7)**

a Mice were scored histopathologically for worst disease present in the cervix and vagina (in parentheses). CIN, cervical intraepithelial neoplasia; VIN, vaginal intraepithelial neoplasia. A two-sided Wilcoxon rank sum test was used to compare overall cervicovaginal disease severity (worst disease in cervix and vagina combined). The only comparisons between mock-infected and MmuPV1-infected groups that reached statistical significance are the following: MmuPV1+E2 versus Mock+E2 (*, *P* = 0.004) and MmuPV1+UV+E2 versus Mock+UV+E2 (**, *P* = 0.001).

10.1128/mBio.00180-19.3 FIG S3 C57BL/6 mice are resistant to MmuPV1-induced carcinogenesis. (A) Disease severity comparison at 4 months postinfection in the female reproductive tract of FVB (*n* = 13) and C57BL/6 (*n* = 7) mice at 4 months after infection with MmuPV1+UV+E2. Overall disease severity was compared using a two-sided Wilcoxon rank sum test, and asterisks indicate *P* ≤ 0.001. (B) H&E and indirect immunofluorescence analysis for L1 (red), K14 (green), and DAPI (blue) on representative samples from FVB and C57BL/6 groups of mice infected with MmuPV1+UV+E2. Download FIG S3, PDF file, 5.6 MB. Copyright © 2019 Spurgeon et al. 2019 Spurgeon et al. This content is distributed under the terms of the Creative Commons Attribution 4.0 International license.

We also compared cervicovaginal disease severity (by combining cervical and vaginal scores) among MmuPV1-infected cohorts of FVB/N mice (Fig. 2D). In the MmuPV1-only cohort, 25% (*n* = 2/8) of mice were disease free and the remaining 75% (*n* = 6/8) developed primarily low- to moderate-grade dysplasia (grade 2). When MmuPV1-infected mice were exposed to UVB radiation (MmuPV1+UV), nearly 80% of mice (*n* = 7/9) developed neoplastic disease, 22% (*n* = 2/9) of mice developed grade 2 dysplasia, and 56% (*n* = 5/9) developed grade 3 dysplastic lesions. The effect of UVB on disease severity was not significant compared to MmuPV1 alone (*P* = 0.11). However, exogenous estrogen treatment significantly increased cervicovaginal disease severity compared to virus alone (MmuPV1+E2 versus MmuPV1 only, *P* = 0.001). All MmuPV1+E2 mice (*n* = 8) developed moderate- to high-grade precancerous lesions. Although UVB radiation alone did not significantly increase overall disease severity in MmuPV1-infected mice, it appeared to synergize with exogenous estrogen treatment to promote SCC development. Nearly 70% of MmuPV1+UV+E2 mice (*n* = 9/13) developed SCC, and the remaining mice developed moderate- to high-grade dysplasias. This increase in cervicovaginal disease severity was highly significant (*P* = 9.53 × 10^−5^) compared to mice infected with MmuPV1 but not treated with UV and E2. The effect of UVB and estrogen appeared to be synergistic, as the severity of disease in MmuPV1+UV+E2 mice was significantly higher than with either factor alone (MmuPV1+UV+E2 versus MmuPV1+UV, *P* = 0.002; versus MmuPV1+E2, *P* = 0.011). These results indicate that MmuPV1 can establish persistent infections in the mucosal epithelia of the female reproductive tract of wild-type, immunocompetent FVB mice that result in SCC by 4 months when infected mice are treated with UV and estrogen.

### Biomarker analysis of MmuPV1-infected reproductive tract epithelia in immunocompetent mice.

**FIG 3:**
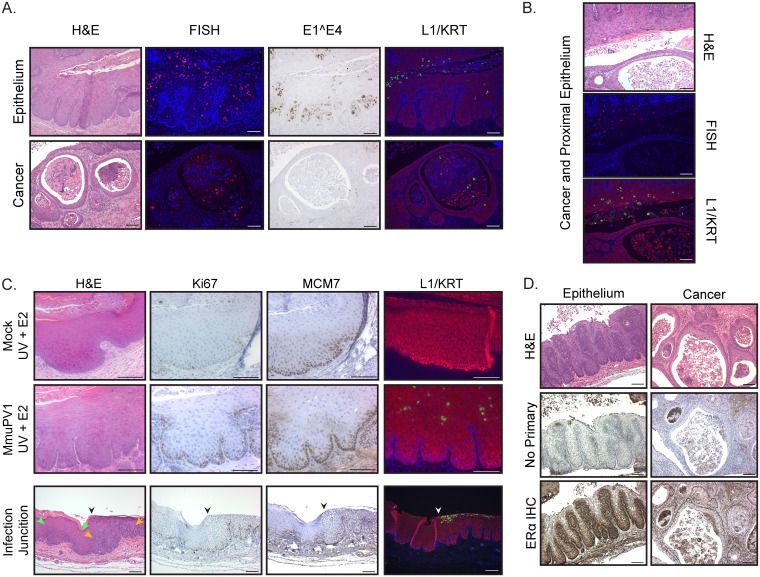
Biomarker analysis of MmuPV1-infected reproductive tract epithelia in immunocompetent mice. (A) Comparison of cervicovaginal epithelia and cancers arising in female FVB mice infected with MmuPV1+UV+E2 at 4 months postinfection. Representative images from the following analyses are shown: H&E staining to observe histopathology, RNAscope to detect MmuPV1 E1^E4 RNA expression (brown signal with hematoxylin counterstain), FISH for MmuPV1 viral DNA (red) with DAPI nuclear stain (blue), and indirect immunofluorescence for MmuPV1 L1 protein (red) with costaining for keratin (KRT) (green) and DAPI nuclear stain (blue). (B) Representative images of H&E, FISH for MmuPV1 DNA, and L1/KRT indirect immunofluorescence showing a cervicovaginal cancer and proximal epithelium in a mouse infected with MmuPV1+UV+E2. (C) Immunohistochemical analysis of Ki67 and MCM7 (brown signal and hematoxylin counterstain) and indirect immunofluorescence analysis for MmuPV1 L1 protein (red), K14 (green), and DAPI (blue) of cervicovaginal epithelia in Mock+UV+E2 and MmuPV1+UV+E2 mice (top and middle panels, respectively). The same biomarker analyses performed on tissues with a junction (indicated by black/white arrowheads) between uninfected (green arrows) and MmuPV1-infected (orange arrows) epithelia present in an MmuPV1+UV-infected mouse are shown in the bottom panel. (D) Immunohistochemistry analysis for ERα in dysplastic cervicovaginal epithelium and cancers in MmuPV1+UV+E2-infected mice at 4 months postinfection. Corresponding H&E-stained sections and no-primary-antibody controls are also shown. All scale bars = 100 μm.

Focusing on the MmuPV1+UV+E2 cohort, where the most severe disease developed, we analyzed markers of MmuPV1 infection. We found MmuPV1 DNA, E1^E4 transcript, and L1 capsid protein expression both in the cervicovaginal epithelia and in cancers (Fig. 3A). While markers of MmuPV1 virus infection were detected in cancers, their expression was frequently lower than in nontumor epithelia even when regions of productively infected epithelia were present within close proximity to the tumor (Fig. 3B). We have previously found that high-risk HPV induces markers of DNA synthesis (Ki67) and E2F-dependent gene expression (MCM7) in suprabasal cells throughout all layers of the stratified squamous epithelium specifically due to the ability of HPV E7 to degrade pocket proteins (38, 39). We analyzed reproductive tracts where epithelia showed histological hallmarks of virus infection for Ki67 and MCM7 (Fig. 3C). To assess virus-induced changes at this interface, we further analyzed the junctions between regions with normal histopathology and those with histopathological features consistent with virus infection. Qualitatively, we saw increased numbers of Ki67-positive basal cells in infected epithelia compared to controls. However, there was a slight increase in both Ki67 and MCM7-positive cells in the parabasal and suprabasal layers. In contrast to our findings with HPV16, we did not observe Ki67- or MCM7-positive cells throughout the full thickness of the MmuPV1-infected stratified epithelia under any experimental condition.

In human cervical cancers, epithelial expression of the estrogen receptor ERα is progressively lost during the course of HPV-induced neoplastic progression (40). To determine the status of ERα expression in MmuPV1-induced disease, we performed immunohistochemistry for ERα on MmuPV1-infected reproductive tracts (Fig. 3D). Expression of ERα persisted both in MmuPV1-infected epithelia and in cancer epithelial cells, consistent with estrogen-treated HPV16 transgenic mice, where ERα expression is retained (33).

### Extended duration of infection with MmuPV1 or MmuPV1+E2 is sufficient to drive carcinogenesis.

**FIG 4:**
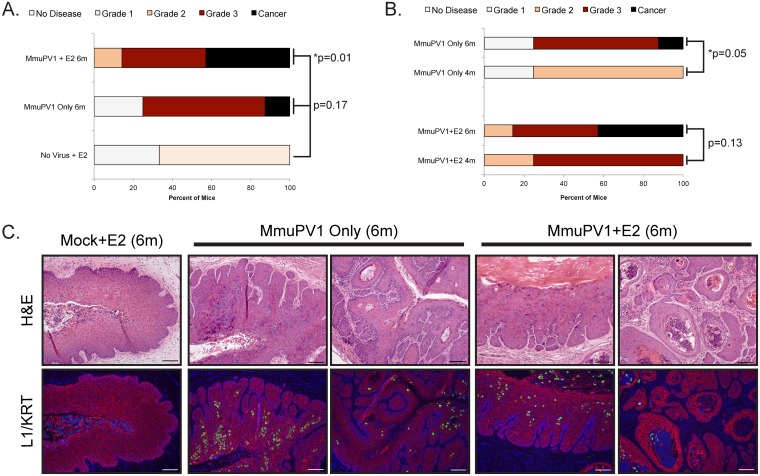
Extended-duration MmuPV1 infection, alone or with estrogen treatment, is sufficient to drive carcinogenesis in immunocompetent mice. (A) Disease severity in cohorts of MmuPV1-infected mice as determined by histopathological analysis. Control Mock + E2 6m (*n* = 3) mice were compared to MmuPV1 Only 6m (*n* = 8; *P* = 0.17) and MmuPV1 + E2 6m (*n* = 7; *P* = 0.01) mice using a two-sided Wilcoxon rank sum test. *P* values are indicated, and an asterisk indicates statistical significance of ≤0.05. (B) Disease severity is compared between MmuPV1-only infections at 4 months (*n* = 8) and 6 months (*n* = 8) postinfection, and MmuPV1 + E2 4m (*n* = 8) and MmuPV1 + E2 6m (*n* = 7) using a two-sided Wilcoxon rank sum test. *P* values are indicated, and an asterisk indicates statistical significance of ≤0.05. (C) H&E and immunofluorescence analysis for MmuPV1 L1 (red), keratin (green), and DAPI staining for nuclei (blue) showing representative neoplastic disease at 6 months postinfection. All scale bars = 100 μm.

Approximately 75% of MmuPV1+E2 mice developed grade 3 cervicovaginal disease at 4 months postinfection (Fig. 2D; Table 1). We previously demonstrated that HPV16 transgenic mice develop SCC when treated with estrogen for 6 months (31). We therefore tested the hypothesis that extended treatment with estrogen increases the severity of tumorigenesis in MmuPV1-infected mice. Mice were infected with MmuPV1, followed by 6 months of exogenous estrogen treatment. Groups of mock-infected, estrogen-treated mice and mice infected with only MmuPV1 for 6 months were included as controls. Overall disease severity in MmuPV1+E2 (6 months) was significantly increased over mock-infected controls (*P* = 0.01; Fig. 4A). Although overall disease severity did not significantly increase in 6-month versus 4-month estrogen-treated MmuPV1-infected mice (*P* = 0.13; Fig. 4B), approximately 43% (*n* = 3/7) of MmuPV1+E2 (6 months) developed SCC, whereas no MmuPV1+E2 (4 months) mice had cancer. Therefore, 6 months of exogenous estrogen treatment was sufficient to promote carcinogenesis in MmuPV1-infected mice. Surprisingly, mice infected with MmuPV1 alone for 6 months also developed high-grade disease and SCC (Fig. 4A). Allowing MmuPV1 infection to proceed for 6 months instead of 4 months significantly increased the severity of disease (*P* = 0.05; Fig. 4B), and SCC was observed in one mouse (*n* = 1/8, 12.5%). High-grade lesions and SCC present in MmuPV1-only and MmuPV1+E2 mice at 6 months postinfection were associated with productively infected epithelia (Fig. 4C). These results indicate that prolonged MmuPV1 infection can promote cervicovaginal carcinogenesis in immunocompetent mice. Furthermore, extended estrogen treatment facilitates malignant progression in MmuPV1-infected mice.

## DISCUSSION

Here we report a novel infection model for papillomavirus-mediated neoplastic progression in the murine female reproductive tract with MmuPV1 (Fig. 1). We confirmed that infection of the mucosal epithelia of the female reproductive tract in immunodeficient (*FoxN1^nu/nu^*) mice with MmuPV1 leads to neoplastic disease and malignant progression (see Fig. S1 in the supplemental material), including progression to SCC. Importantly, we demonstrate that wild-type, immunocompetent mice on the FVB genetic background are susceptible to MmuPV1-associated neoplastic disease and carcinogenesis in the female reproductive tract (Fig. 2; Table 1). Additionally, we report that two cofactors involved in papillomavirus-mediated disease previously identified by our laboratory, UV radiation in the context of MmuPV1 infection (24) and the female hormone estrogen in the context of HPV16 transgenic mice (31–33), contribute to MmuPV1 infection and malignant progression in this cervicovaginal infection model (Fig. 2; Table 1). Unlike cutaneous epithelia, where MmuPV1 fails to cause robust phenotypic disease in wild-type, immunocompetent mice (24), we found that female reproductive tract mucosal epithelia infected with the same dose of MmuPV1 can develop low to moderate levels of dysplastic disease by 4 months postinfection (Fig. 2) and high-grade lesions and SCC by 6 months postinfection (Fig. 4). Estrogen significantly increased disease severity in MmuPV1-infected mice and cooperated with UV to promote cervicovaginal carcinogenesis at 4 months postinfection. However, extended treatment with estrogen alone was sufficient to drive carcinogenesis in MmuPV1-infected mice (Fig. 4). Biomarker analysis indicated that common readouts of high-risk HPV infection, such as DNA synthesis and E2F-dependent gene expression, are not similarly increased in MmuPV1-infected cervicovaginal epithelia (Fig. 3), thus identifying interesting and likely informative areas of molecular virology for future study.

Recently, Cladel et al. reported a model in which MmuPV1-infected outbred heterozygous nude mice (*FoxN1^nu/+^*) develop carcinoma *in situ* in the female reproductive tract (28). Although the investigators describe *FoxN1^nu/+^* mice as immunocompetent, there is evidence that *FoxN1* haploinsufficiency affects the immune system. *FoxN1* gene dosage is important for postnatal thymus homeostasis, and *FoxN1^nu/+^* mice exhibit several postnatal defects such as reduced weight and thymus size, reduction of thymocyte numbers, and impaired thymic epithelial cell (TEC) differentiation and proliferation (41, 42). Moreover, expression of a group of genes that regulate TEC function is sensitive to *FoxN1* gene dosage (43). *FoxN1* haploinsufficiency led to reduced expansion of leukemic T cells (44). Therefore, the effects of *FoxN1* haploinsufficiency on thymic organogenesis and function should be critically considered given the correlation between T-cell status and MmuPV1 pathogenesis (25, 26). Our study demonstrating MmuPV1-induced cervicovaginal disease in FVB mice represents the first true example of *de novo* papillomavirus-mediated carcinogenesis in wild-type mice that are genetically immunocompetent.

Persistent infections in HPV-infected women significantly increase the risk for high-grade disease development (45). A notable aspect of this model is that the mucosal cervicovaginal epithelium of FVB mice supported persistent MmuPV1 infection, and persistent infection correlated with the severity of subsequent disease (Fig. 2). Some mice within the MmuPV1 and MmuPV1+UV groups did not establish persistent infections (Fig. 2B), likely contributing to their overall disease severity being the lowest among the four treatment groups (Fig. 2D). On the other hand, all MmuPV1-infected mice treated with estrogen, either alone or in combination with UV, established persistent infections. The level of exogenous estrogen administered to mice in our study can induce persistent estrus (30), leading to persistent proliferation of epithelial cells (46), which likely provides a more permissive environment for MmuPV1 to establish its life cycle. This finding is consistent with the observation that MmuPV1 viral copy numbers are highest during estrus phase in immunodeficient mice (21). We therefore hypothesize that estrogen facilitates the establishment and/or persistence of MmuPV1 infection in the female reproductive tract.

Viral persistence was also influenced by pretreatment with medroxyprogesterone acetate and genetic background. Mice that were not pretreated with medroxyprogesterone acetate were less likely to establish persistent MmuPV1 infections and consequently developed lower-grade disease (Fig. S2). One potential explanation for this finding is that medroxyprogesterone acetate induces diestrus, thinning the stratified epithelium and increasing access to basal cells that support establishment of infections. This compound also has immunosuppressive qualities that are known to increase other microbial infections (47, 48). Since the immune environment is a critical factor in MmuPV1 cutaneous pathogenesis (24–27), it is possible that alteration of immune responses by medroxyprogesterone acetate facilitates MmuPV1 infection in the female reproductive tract. We also found that mice on the C57BL/6 genetic background are resistant to MmuPV1-induced cervicovaginal disease (Fig. S3). This is consistent with other reports that C57BL/6 mice are susceptible to MmuPV1-induced cutaneous disease only upon complete T-cell depletion or UVB irradiation (22, 24, 25). While others have detected L1-positive cells in asymptomatically infected skin of C57BL/6 mice (25), we did not detect L1-positive cells in the reproductive tracts of MmuPV1+UV+E2-infected C57BL/6 mice at 4 months postinfection (Fig. S3). Therefore, different genetic backgrounds and tissue sites exhibit variable susceptibility to MmuPV1 infection. Asymptomatic infections may exist in our model below the limit of detection, and longitudinal studies and additional readouts are required to characterize more fully MmuPV1 persistent infections and the contribution of other factors to this process.

We observed a significant effect of UVB radiation on cervicovaginal disease in MmuPV1-infected mice: MmuPV1+UV+E2 mice developed high-grade precancerous lesions and SCC by 4 months postinfection (Fig. 2). We have recently reported that UV radiation (UVR) makes wild-type immunocompetent mice highly susceptible to MmuPV1-induced cutaneous disease by causing systemic immunosuppression (24). It is possible that UV promotes high-grade cervicovaginal disease by a similar mechanism, but one that also requires the action of estrogen. Recent epidemiological studies indicate a correlation between cervical cancer incidence and UV exposure in Caucasian females (49). UV radiation can also affect both skin-associated infections, such as those caused by herpes simplex virus (HSV-1), and others that are systemic and are noncutaneous (reviewed in reference 50). Therefore, the potential role of UV in papillomavirus-mediated cervicovaginal disease is an area that warrants further investigation and is an area that can be addressed using our immunocompetent MmuPV1 infection model.

The work of our lab and others has identified estrogen as a necessary cofactor required for cervical carcinogenesis in HPV16 transgenic mice (29, 31, 33), and we have demonstrated that this hormone contributes to the onset, maintenance, and malignant progression of HPV16-induced cervical cancer (32). Data presented here support and further strengthen the evidence arising from studies of HPV16 transgenic mice. Estrogen treatment increased the likelihood of establishing a persistent infection and significantly increased the incidence of high-grade precancerous lesions in MmuPV1-infected mice at 4 months postinfection (Fig. 2), and 6 months of estrogen treatment was sufficient to promote carcinogenesis in MmuPV1-infected animals (Fig. 4). These data suggest that estrogen not only plays a role in malignant progression, as observed in HPV transgenic mice, but could also potentiate viral infection and persistence. This is the first demonstration of estrogen as a critical cofactor in promoting neoplastic progression in a natural infection model of papillomavirus-mediated cervicovaginal disease. In future studies, we will employ this model to further explore several preexisting areas of estrogen-related research, such as testing whether antiestrogen treatments can prevent and treat cervical cancer in a *de novo* infection setting, as we have shown previously in HPV16 transgenic mice (51). In HPV16 transgenic mice, we found that the effect of estrogen on cervical carcinogenesis is mediated by estrogen signaling in the stroma (40, 52) and that E6 and E7 oncogenes profoundly affect gene expression in the cervical microenvironment (53). Therefore, it will be important to determine if similar changes occur in a natural infection model.

We found reduced expression of viral infection markers in tumors compared to nearby MmuPV1-infected epithelia (Fig. 3). It is possible that MmuPV1 integrates into the host genome, leading to persistent viral oncogene expression as seen with high-risk mucosal HPVs (54). It remains to be determined whether MmuPV1 integrates into the host genome in the cervicovaginal mucosal epithelia in immunocompetent mice, but our RNA-seq analysis of MmuPV1-induced skin papillomas arising in nude mice indicates the presence of virus-host chimeric reads consistent with viral genome integration (unpublished data). Therefore, MmuPV1 infection models open the door to studying papillomavirus viral genome integration *in vivo* and its association with disease. Some rodent papillomavirus proteins, including MmuPV1, have different expression patterns, structural motifs, and binding partners than HPVs (15, 55). Several dependable markers of HPV oncogene function, such as increased suprabasal DNA synthesis and E2F-dependent gene expression, were not markedly affected in MmuPV1-infected cervicovaginal epithelium (Fig. 3C). While some studies have been initiated (55, 56), the biochemical properties and molecular functions of MmuPV1 E6 and E7 need to be further explored, as it is still unclear whether and/or how well MmuPV1 viral oncogenes mirror HPV oncogene functions, although both clearly have oncogenic potential.

In summary, we have developed a novel preclinical model of papillomavirus-induced cervicovaginal disease by infecting the female reproductive tracts of wild-type mice with MmuPV1. By establishing our model in immunocompetent mice, this system provides a physiologically relevant platform that more accurately models several aspects of papillomavirus-induced disease in the female reproductive tract that can be used to advance several areas of basic and translational research in the papillomavirus field.

## MATERIALS AND METHODS

### Animals.

The following mice were included in this study: 6- to 8-week-old immunodeficient athymic *FoxN1^nu^* mice (Hsd:Athymic Nude-FoxN1^nu^; Envigo, Somerset, NJ) and immunocompetent, wild-type *FVB/N* mice (Taconic Biosciences, Albany, NY) and C57BL/6 mice (Jackson Laboratory, Bar Harbor, ME). All animal experiments were performed in full compliance with standards outlined in the *Guide for the Care and Use of Laboratory Animals* (57) by the Laboratory Animal Resources (LAR) as specified by the Animal Welfare Act (AWA) and Office of Laboratory Animal Welfare (OLAW) and approved by the Governing Board of the National Research Council (NRC). Mice were housed at the McArdle Laboratory Animal Care Unit in strict accordance with guidelines approved by the Association for Assessment of Laboratory Animal Care (AALAC), at the University of Wisconsin Medical School. All protocols for animal work were approved by the University of Wisconsin Medical School Institutional Animal Care and Use Committee (IACUC; protocol number M005871).

### Tissue procurement, processing, and histopathological analysis.

Reproductive tracts were harvested, fixed in 4% paraformaldehyde, and paraffin embedded. Serial sections (5 μm) were cut, and every 10th section was stained with H&E and evaluated by histopathological analysis and scored for worst disease by a trained pathologist in the Department of Pathology and Laboratory Medicine (University of Wisconsin School of Medicine and Public Health). The scoring system is described in detail in Results. Images of H&E-stained cervical tumors and epithelia were captured using a Zeiss AxioImager M2 microscope and AxioVision software version 4.8.2 (Jena, Germany). Full-slide scans of H&E-stained slides were performed using an Aperio ScanScope XT slide scanner using a 20×/0.75 Plan Apo objective.

### MmuPV1 infection of female reproductive tracts.

Mice were infected with MmuPV1 virus stock generated by isolating virions from papillomas from infected *FoxN1^nu/nu^* mice as described previously (24). The female reproductive tract infection strategy was adapted from methods described previously (34, 35). Briefly, mice were injected subcutaneously with 3 mg medroxyprogesterone acetate (Amphastar Pharmaceuticals, Rancho Cucamonga, CA) 4 days prior to MmuPV1 infection to induce diestrus. On the day of the infection, mice were pretreated vaginally with 50 µl Conceptrol (Options; catalog no. 247149) containing 4% nonoxynol-9 to induce chemical injury to the cervicovaginal epithelium (34). At 4 h after treatment with Conceptrol, 10^8^ VGE MmuPV1 virions suspended in 25 µl 4% carboxyl methylcellulose (Sigma; catalog no. C4888) were delivered intravaginally. All treatments were performed while mice were anesthetized with 5% isoflurane.

### Estrogen treatment.

Treatment with exogenous estrogen was performed as described previously (29, 32). Briefly, female mice were anesthetized with 5% isoflurane, and a continuous-release estrogen (E2) tablet (17β-estradiol; 0.05 mg/60 days; Innovative Research of America, Sarasota, FL) was inserted subcutaneously in the shoulder fat pads of the dorsal skin. For those mice receiving estrogen, treatment began 5 days following MmuPV1 infection. A new tablet was inserted every 2 months as needed.

### UVB radiation.

Animals were exposed to a single dose of UVB at 1000 mJ/cm^2^ as described previously (24, 58). UVB was administered using a custom-designed research irradiation unit (Daavlin, Bryan, OH) with lamps controlled using Daavlin flex control integrating dosimeters.

### Vaginal lavage and detection of MmuPV1 by PCR.

The method for detecting MmuPV1 DNA by PCR in vaginal lavage samples was modified from that described in Hu et al. and Cladel et al. (21, 28). Briefly, 25 µl of sterile PBS was introduced intravaginally with a pipette tip, rinsing 4 to 5 times. The vaginal lavage samples were stored at −20°C, and then DNA was isolated using spin columns (DNeasy Blood and Tissue kit; Qiagen catalog no. 69506; Hilden, Germany). Eluted DNA was analyzed by PCR using primers specific to the MmuPV1 E2 gene, MmuPV1_E2_1 (5′-GCCCGAAGACAACACCGCCACG-3′) and MmuPV1_E2_2 (5′-CCTCCGCCTCGTCCCCAAATGG-3′), and analyzed using agarose gel electrophoresis. The primers for the p53 gene were as follows: p53-1 (5′-TATACTCAGAGCCGGCCT-3′), p53-2 (5′-ACAGCGTGGTGGTACCTTAT-3′), and p53-3 (5′-TCCTCGTGCTTTACGGTATC-3′).

### FISH for MmuPV1 DNA.

MmuPV1 DNA fluorescent *in situ* hybridization (FISH) was performed as described previously (58, 59). This protocol has been adapted from a DNA FISH protocol used to detect Epstein-Barr virus (EBV) DNA in monolayer cells and is described in detail at https://mcardle.wisc.edu/sugden/protocols.html.

### MmuPV1 L1-cytokeratin dual immunofluorescence and immunohistochemistry.

A tyramide-based signal amplification (TSA) method was developed to detect MmuPV1 L1 (60). A detailed protocol is available at https://doi.org/10.17504/protocols.io.i8cchsw. For immunohistochemical staining, tissue sections were deparaffinized with xylenes and rehydrated with graded ethanol. Heat-induced antigen retrieval was performed in 0.01 M citrate buffer, pH 6.0. Antibodies against the following proteins were used: estrogen receptor alpha (ERα) (Santa Cruz, Dallas, TX), Ki67 (Dako, Carpinteria, CA), and minichromosome maintenance protein 7 (MCM7) (Thermo Scientific, Fremont, CA). Biotinylated horse anti-mouse/rabbit IgGs (Vector Laboratories, Burlingame, CA) were used as secondary antibodies. Proteins were visualized with 3,3′-diaminobenzidine (Vector Laboratories), and tissues were counterstained with hematoxylin.

### RNA *in situ* hybridization.

MmuPV1 viral transcripts were detected using RNAscope 2.5 HD Assay-Brown (Advanced Cell Diagnostics, Newark, CA) according to the manufacturer’s instructions (61) with probes specific for MmuPV1 E1^E4 (catalog no. 473281). Tissue sections were treated following protease treatment and prior to probe hybridization with 20 units of DNase I (Thermo Fisher Scientific; catalog no. EN0521) for 30 min at 40°C. Slides were counterstained with hematoxylin before mounting and coverslipping.

### Fluorescence image acquisition.

High-resolution wide-field fluorescent images were acquired using a Leica SP8 3X STED microscope (61) by means of a 20× lens objective (specifications: HC PL APO 20×/0.75 CS2, dry) and the LAS-X suite (version 2.0.1). All other images were captured using a Zeiss AxioImager M2 microscope and AxioVision software version 4.8.2 (Jena, Germany).

### Statistical analysis.

All statistical analyses were performed using MSTAT statistical software version 6.4.2 (http://www.mcardle.wisc.edu/mstat; last accessed on 19 October 2018).
