# Supplementary figures and images for: A Novel In Vivo Infection Model To Study Papillomavirus-Mediated Disease of the Female Reproductive Tract
Source: mBio. 2019 Mar 5;10(2):e00180-19. doi: 10.1128/mBio.00180-19 (PMC6401479; doi:10.1128/mBio.00180-19)

Figure S1

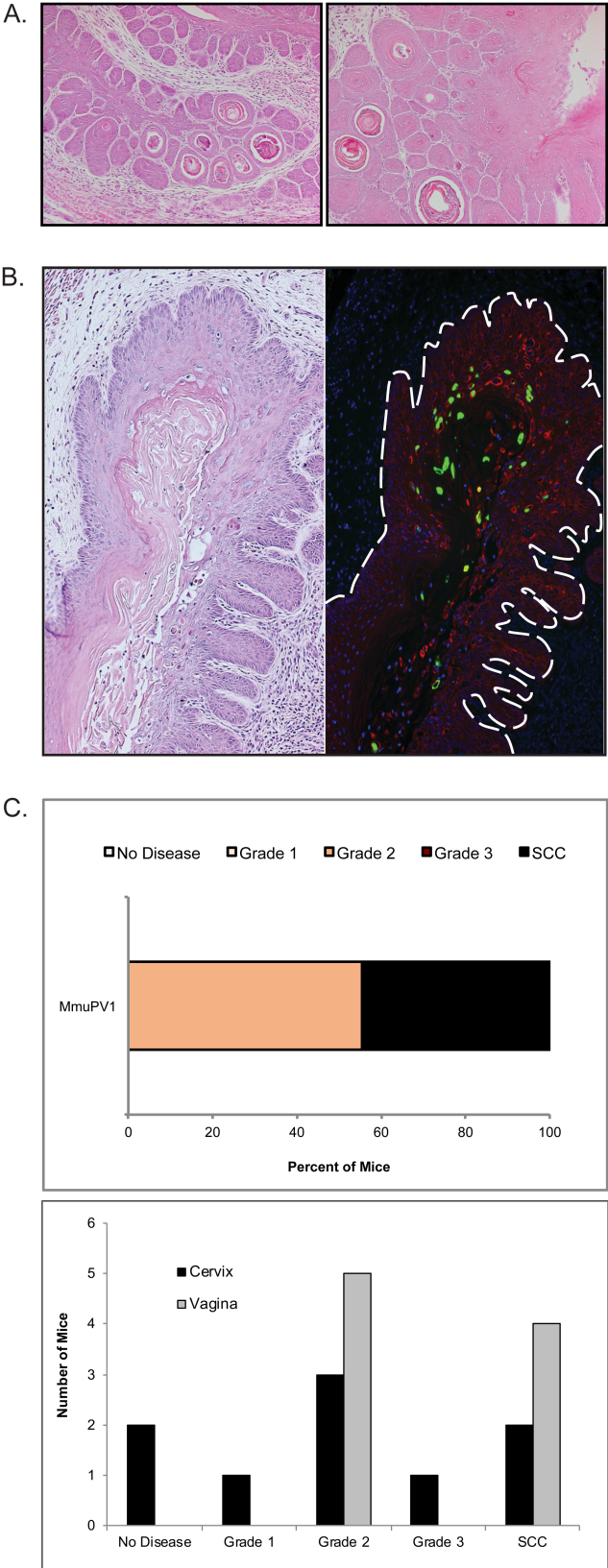

Supplement: FIG S1 [file mBio.00180-19-sf001.pdf]

Figure S2

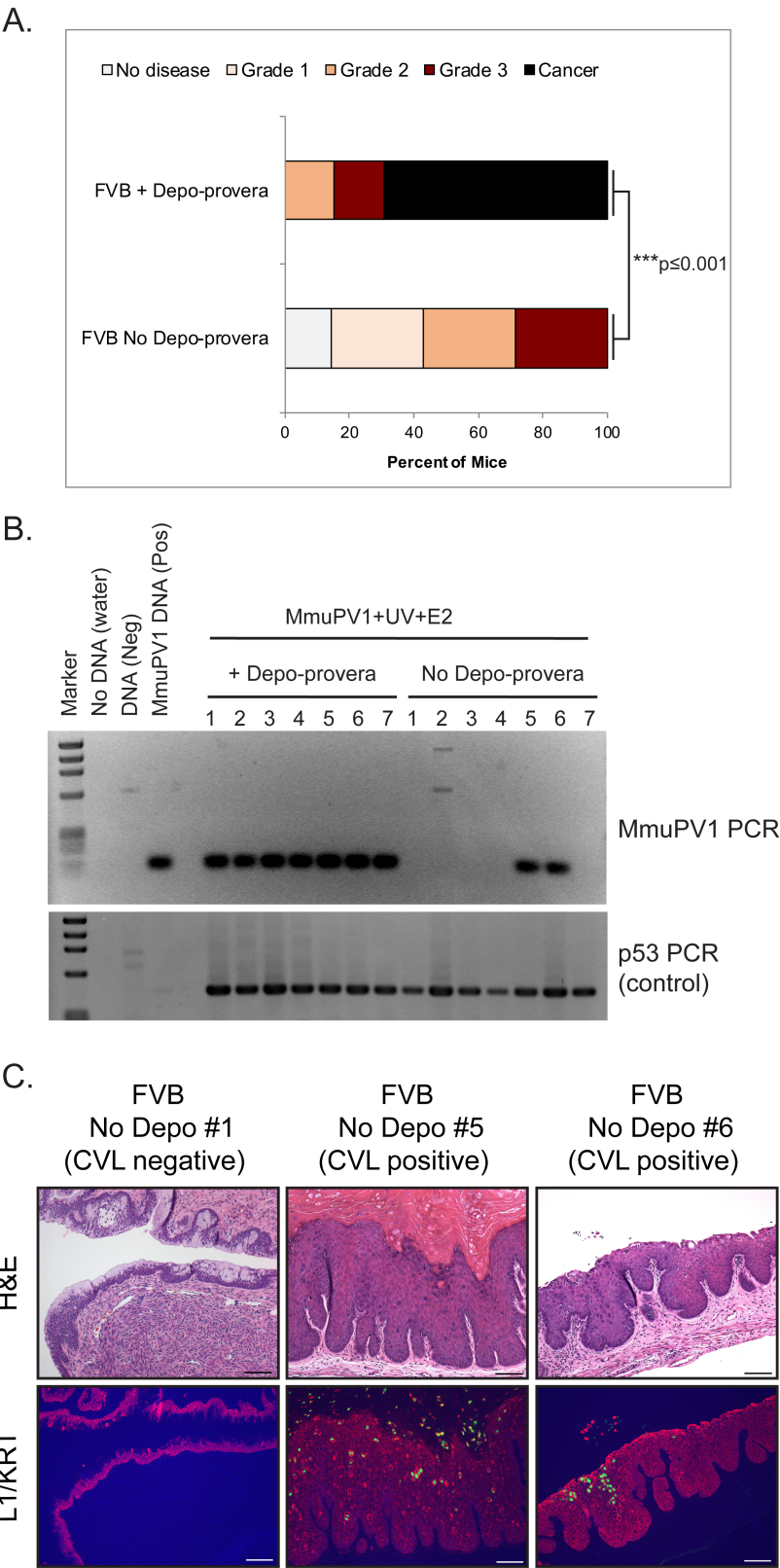

Supplement: FIG S2 [file mBio.00180-19-sf002.pdf]

Figure S3

A.

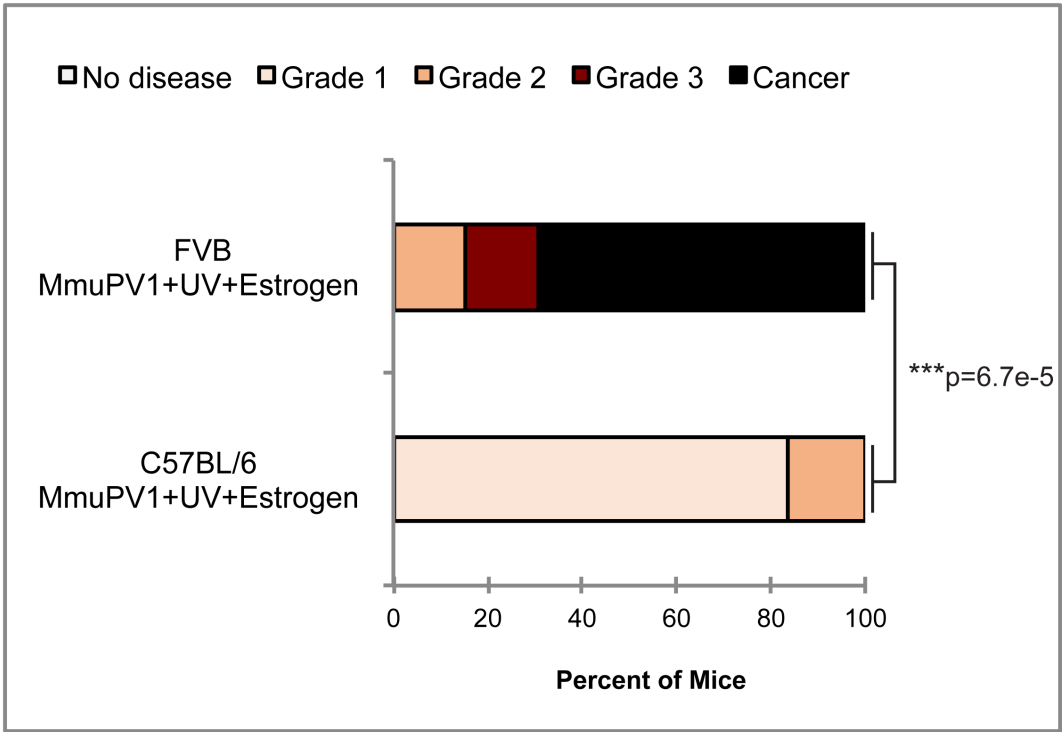

B.

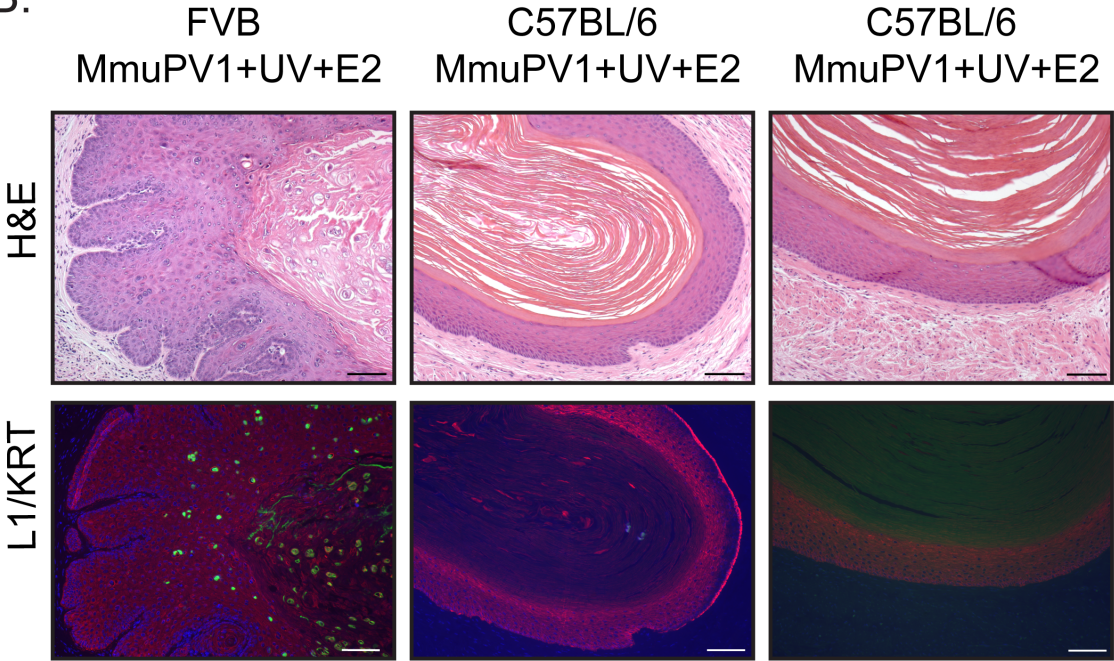

Supplement: FIG S3 [file mBio.00180-19-sf003.pdf]
